# The effects of biogenic amines in Chinese Huangjiu on the behavior of mice and hangover headache‐related indices

**DOI:** 10.1002/fsn3.3016

**Published:** 2022-08-17

**Authors:** Wenmei Zhao, Zukang Fu, Xin Wang, Qingzhong Mao, Chunguang Luan, Shanbin Chen, Fengjie Zhang, Jiajun Yu, Yiping Yao, Yishu Li, Feike Hao, Deliang Wang, Nan Li, Jie Huangfu, Chengtao Wang

**Affiliations:** ^1^ College of Life Science and Technology Guangxi University Nanning China; ^2^ China National Research Institute of Food and Fermentation Industries Beijing China; ^3^ Kuaijishan Shaoxing Rice Wine Co. Ltd. Shaoxing China; ^4^ School of Food Science and Engineering Beijing Technology and Business University Beijing China

**Keywords:** 5‐HT, biogenic amines, headache, histamine, Huangjiu

## Abstract

Huangjiu (Chinese rice wine) is a popular and traditional alcoholic beverage in China; however, the consumption of Huangjiu readily results in hangover symptoms. The aim of this study was to identify the main components associated with behavioral inhibition, headache, and the relevant mechanisms by using a mice hangover model. The results of an open‐field experiment revealed that the key biogenic amine associated with mice behavior was histamine, which inhibited the behavior activity of mice in a dose‐dependent manner. Moreover, histamine treatment decreased the levels of serotonin (5‐HT) and 5‐hydroxyindole acetic acid. In addition, the levels of dopamine and nitric oxide, which are associated with migraine, increased in the brain tissue of mice. In addition, the expression of receptor genes of 5‐HT, including *Htr1a*, *Htr1f*, and *Htr2c*, is essential in regulating various behaviors and mental activities. In conclusion, the present study demonstrated that histamine is a key component in Huangjiu, and it is related to hangover symptoms by affecting the level of 5‐HT and its receptors.

## Graphical abstract

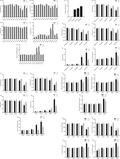

## 1 INTRODUCTION

Huangjiu (Chinese rice wine) is a traditional brewed alcoholic beverage originating in China, and it is known as one of the world's three ancient wines, together with wine and beer (Xu et al., 2010). Owing to its unique flavor, taste, and high nutritional value, Huangjiu is popular with the majority of consumers. It is also called “liquid cake” because of its sweet flavor (Liu, 2017; Wang et al., 2011). A variety of functional substances with special biological activity have been detected in Huangjiu, such as functional oligosaccharides, polyphenols, monacolin K, and λ‐aminobutyric acid (Wang et al., 2014). These compounds exhibit physiological effects on human health, including blood pressure and cholesterol reduction, as well as antiaging and immune regulation effects (Liu et al., 2015).

However, compared with Baijiu, a traditional Chinese distilled spirit, Huangjiu readily causes hangover symptoms, such as dizziness, headache, flushed face, behavior disorder, and inattentiveness (Shan et al., 2016; Zhang et al., 2015). Recent studies have shown that biogenic amines (BAs), fusel oil, and acetaldehyde were the main causes of dizziness and headache (Panconesi, 2008; Wöber & Wöber‐Bingöl, 2010). BAs are nitrogen‐containing organic compounds with low molecular weight, and they are widely found in beverages and foods (Lu et al., 2007). Types of BAs mainly include histamine, cadaverine, tyramine, 2‐phenylethylamine, and putrescine (Kim et al., 2011). Some cause headache‐related symptoms (Wöber & Wöber‐Bingöl, 2010). Among them, histamine has an inhibitory effect on the central nervous system and can cause dizziness, headache, and drowsiness. The headache caused by histamine is diagnosed as a migraine by the International Headache Association (Olesen et al., 2004). However, the behavioral indicators of various hangover symptoms associated with BAs require further study.

Serotonin (5‐HT) neurons are mainly distributed in the medulla oblongata, pons, and midbrain of the central nervous system. Changes in the content of 5‐HT could lead to changes in physiological functions, such as appetite, sleep, and sexual behavior (Zhong et al., 2019). The subpopulations of 5‐HT neurons co‐express different neurotransmitters and neuromodulators including dopamine (DA) (Monti, 2011). In a recent study, neurotransmitters related to headaches were identified, including 5‐HT, 5‐hydroxyindole acetic acid (5‐HIAA), and DA (Han et al., 2004; Wang et al., 2018). Moreover, nitric oxide (NO) is a key factor that contributes to the state of a migraine (Olesen, 2008). The biosynthesis of 5‐HT is regulated by various extracellular signaling molecules and intracellular transcription factors (Wang et al., 2018). Therefore, the above substances can trigger migraines; however, the interactions between them remain unclear.

At present, hangover‐induced dizziness, headache, and movement imbalance have been largely underexplored. This research sheds new light on BAs found in Huangjiu by using an alcohol consumption mice model. The behavioral indicators of mice were analyzed to find out which types of BAs could cause various hangover symptoms. In addition, the content of 5‐HT, 5‐HIAA, DA, and NO in brain tissue was also analyzed to explore the association between histamine and these substances. The key genes of the 5‐HT generation pathway were explored to reveal the mechanism of migraines. This work provides a foundation for improving the quality of Chinese Huangjiu.

## 2 MATERIALS AND METHODS

### 2.1 Chemicals and reagents

Four brands of commercial Huangjiu A, B, C, and D (14% vol.) were purchased from a local supermarket in Beijing. The edible alcohol (14% vol.) was purchased from Azelis Co., Ltd. Glucose and maltose were purchased from Yuanye Biotechnology Co., Ltd. Amino acids were purchased from Sigma‐Aldrich. Putrescine, 2‐phenylethylamine, cadaverine, histamine, tyramine, fusel oil, and acetaldehyde were purchased from Banxia Biotechnology Co., Ltd. Ethyl lactate, ethyl acetate, diethyl succinate, vanillic acid, lactic acid, succinic acid, acetic acid, phenylacetaldehyde, furfural, vitamins C, Ca, K, and Mg were purchased from Sinopharm Chemical Reagent Co., Ltd. The 5‐HT, 5‐HIAA, DA, and NO detection kits were purchased from Nanjing Jiancheng Biological Engineering Co., Ltd. A high‐purity total RNA rapid extraction kit was purchased from Beijing Biotech Biotechnology Co., Ltd. A ReverTra Ace® qPCR RT Master Mix kit was purchased from Toyobo Co., Ltd. Real‐time fluorescent quantitative primers were supplied from Beijing Huada Gene Research Center. All other chemical reagents were of analytical grade.

### 2.2 Animals

SPF male C57BL/6 mice (6–8 weeks, 20 ± 2 g) were obtained from Beijing Weitong Lihua Laboratory Animal Technology Co., Ltd. The present study was approved by the Ethics Committee of Joekai Biotechnology Co. Ltd. (SYXK 2021–0025). Mice were raised in the Beijing Food Additive Engineering Technology Research Center. All animals were housed individually in cages under standard laboratory conditions (humidity 40 ± 5%, room temperature 23 ± 2°C, and 12 h light–dark cycle) and were allowed free access to water and food. All procedures were carried out in accordance with the Guidance Suggestions for the Care and Use of Laboratory Animals, enacted by the Ministry of Science and Technology of China.

### 2.3 Experimental design of mice behavior

The postdrinking mice behavior was evaluated in terms of comfortableness. The open‐field test (Paré, 1994) was used to evaluate the exploratory and autonomous behavior of mice in a new environment. The open‐field test device includes an isolator (height of 40 cm and diameter of 50 cm) and a video tracking analysis system. The protocol described in a previously published method was employed, with minor modifications (Wang et al., 2011). The behavior test included learning trials and formal trials. In learning trials, each mouse underwent three daily training trials for a week and then started the formal trials. Behavioral data were recorded by ANY‐maze software, including moving distance, average speed, moving time, stagnation time, and the number of stagnation periods. All mice were sacrificed after trials. Brain samples were collected, put on ice, and stored in liquid nitrogen for biochemical parameter assays. All surgeries were performed under 4% sodium pentobarbital anesthesia.

### 2.4 Influence of different BAs on the behavior of mice

The blended wine is a simulated rice wine prepared by self‐modulation that does not contain any BAs. The production method of blended wine was added some basic substances commonly found in Huangjiu (except BAs) into edible alcohol. The blended wine was used as a base wine for identifying which BA had significant effects on mice compared with the control group. The groups and dosages are shown in Table 1 (EXP 1). Basic substances (carbohydrates, esters, and so on) are shown in Table 2. Behavior changes in mice were recorded by the monitoring method described in Section 2.3 after gavage. The behavioral indicators of each group of mice were measured at 0.5 and 2.5 h after gavage.

**TABLE 1:** Description of the experimental group

Experiment	Description	Groups	Contents	Dosage
EXP 1	Pure water	Blank	–	10 ml/kg (BW)
	Blended wine	Control	–	
	Blended wine + low 2‐phenylethylamine	LPEA	30 mg/L	
	Blended wine + high 2‐phenylethylamine	HPEA	60 mg/L	
	Blended wine + low putrescine	LPUT	30 mg/L	
	Blended wine + high putrescine	HPUT	60 mg/L	
	Blended wine + low cadaverine	LCAD	30 mg/L	
	Blended wine + high cadaverine	HCAD	60 mg/L	
	Blended wine + low histamine	LCAD	30 mg/L	
	Blended wine + high histamine	HCAD	60 mg/L	
	Blended wine + low tyramine	LTYM	30 mg/L	
	Blended wine + high tyramine	HTYM	60 mg/L	
EXP 2	Pure water	Blank	–	
	Huangjiu A	Control	–	
	Huangjiu A + low histamine	LHIM	7 mg/L	
	Huangjiu A + middle histamine	HHIM	35 mg/L	
	Huangjiu A + high histamine	HHIM	69 mg/L	

**TABLE 2:** Basic substances

Basic substance	Classification	Content (mg/L)
Carbohydrate	Glucose	20,000.37 ± 0.50
	Maltose	5000.59 ± 29.31
Esters	Ethyl lactate	298.16 ± 3.17
	Ethyl acetate	33.46 ± 3.92
	Diethyl succinate	6.05 ± 1.53
Carboxylic acid	Lactic acid	4700.96 ± 8.42
	Amber acid	600.40 ± 2.87
	Acetic acid	600.06 ± 0.92
Amino acid	γ‐Aminobutyric acid	2.39 ± 0.15
	3‐Aminopropanoic	577.01 ± 10.27
	Arginine	359.27 ± 9.43
	Glycine	386.46 ± 2.18
	Aspartic acid	268.12 ± 21.78
	Tyrosine	262.22 ± 25.03
	Serine	232.46 ± 8.73
Amino acid	Histidine	90.13 ± 0.99
	Isoleucine	160.62 ± 17.71
	Phenylalanine	278.33 ± 0.97
	Tryptophan	59.92 ± 2.63
	L‐threonine	173.71 ± 11.33
	Valine	204.68 ± 5.66
	Leucine	300.07 ± 10.31
	Proline	339.98 ± 4.46
Fusel oil	β‐Phenethyl alcohol	120.37 ± 8.23
	Isopentanol	264.43 ± 10.02
	Isobutanol	41.11 ± 3.88
	n‐propanol	28.98 ± 1.75
	2‐Methyl‐1‐pentanol	16.62 ± 2.97

### 2.5 Histamine determination in Huangjiu samples

The histamine standard was dissolved with 0.1 mol/L HCl to prepare a 10‐ml standard solution. The internal standard solution was prepared with a 1,7‐diaminoheptane standard and 0.1 mol/L hydrochloric acid solution. One milliliter of Huangjiu sample was pipetted into a 15 ml centrifuge tube. Then, 25 μl internal standard solution, 200 μl saturated sodium bicarbonate solution, 100 μl sodium hydroxide solution (2 mol/L), and 2 ml derivatization with DNS‐Cl were added to the tube. The mixture was vortexed for 1 min, and the tube was placed in a 70°C water bath for derivatization for 10 min. Then, 100 μl ammonia water was added to the tube to terminate the reaction. Separation was performed on an Agilent C18 column (4.6 mm × 150 mm, 5 μm). The column temperature was 35°C, and the injection volume was 50 μl. Mobile phase A consisted of 90% acetonitrile, and mobile phase B was a mixture of 0.01 mol/L ammonium acetate solution and 0.1% acetic acid. Chromatography was performed with an ultraviolet detector at 254 nm. The sample was kept in the dark for 10 min, passed through a 0.22 μm filter membrane into a sample vial, and analyzed by RP‐HPLC, as described by Zhong et al. (2012).

### 2.6 Effects of different brands of Huangjiu samples on mice behavior

Huangjiu A was used as a base wine. The groups and dosage are shown in Table 1 (EXP 2). Behavior changes in mice were recorded by the monitoring method described in Section 2.3 after gavage.

### 2.7 Effect of histamine concentration on mice behavior in group A

A total of 60 mice were randomly divided into five experimental groups, including the blank group (pure water), control group (Huangjiu A), low histamine group (A + 7 mg/L histamine), middle histamine group (A + 35 mg/L histamine), and high histamine group (A + 69 mg/L histamine). All mice in the five groups received Huangjiu at a dosage of 10 ml/kg (BW) via oral gavage.

### 2.8 Content of 5‐HT, 5‐HIAA, DA, and NO in brain tissue

The 5‐HT, 5‐HIAA, DA, and NO contents were detected using commercial kits, and we strictly followed the manufacturers' instructions.

### 2.9 Quantitative reverse‐transcription polymerase reaction (qRT‐PCR) analysis of the expression of *Htr1a*, *Htr1f,* and *Htr2c*

RNA was isolated from brain tissue with a high‐purity total RNA rapid extraction kit. Then, cDNA was obtained using a ReverTra Ace® qPCR RT Master Mix kit and analyzed via a qRT‐PCR system (Bio‐Rad, CFX96). *β‐actin* was used as an internal reference in this experiment, and the related gene expressions of *Htr1a*, *Htr1f*, and *Htr2c* were measured according to the 2^*−ΔΔ*Ct^ formula. The primer sequences used in this study are listed in Table 3.

**TABLE 3:** The primer sequences

Gene	Sequence 5′–3′	
	FW	RV
*Htr1a*	ATTAACTGGCTAGGCTAC	GCACTTGATGATCTTCTTAA
*Htr1f*	TTGAATACGCCAGGAAGA	ATAGAGATGAACACAGATATAACC
*Htr2c*	AAGTGTTCGTGAATAATAC	CGTTAAGAAGTAGGTGAT
*β‐Actin*	GGCTGTATTCCCCTCCATCG	CCAGTTGGTAACAATGCCATGT

### 2.10 Statistical analysis

All the statistical analyses were performed via SPSS version 18.0 software. Significant differences among groups were evaluated by one‐way analysis of variance (ANOVA). *p* < .05 was considered statistically significant.

## 3 RESULTS

### 3.1 Influence of BAs on mice behavior

**FIGURE 1:**
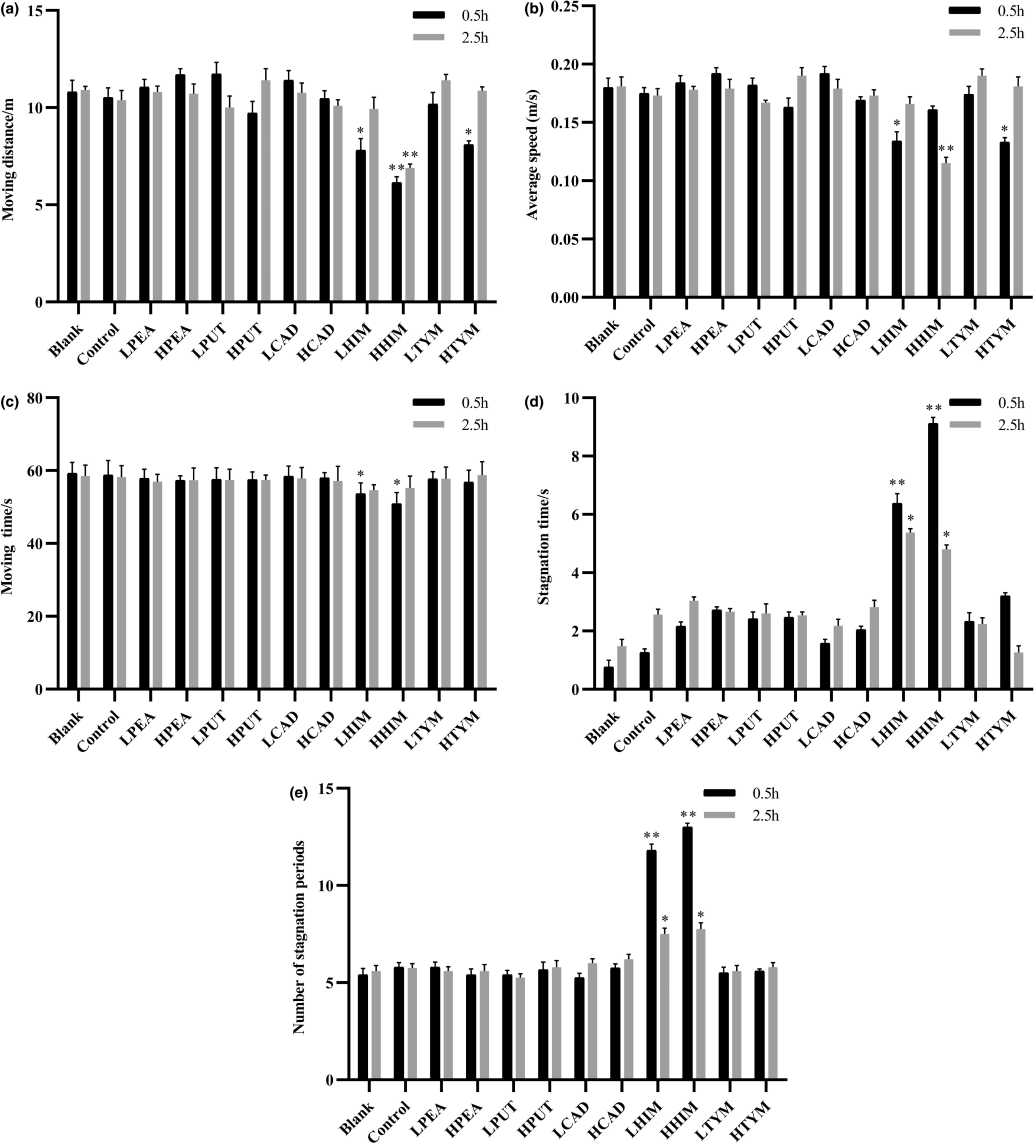
Influence of BAs on mice behavior in treatment groups. (a) Moving distance of mice. (b) Average speed of mice. (c) Moving time of mice. (d) Stagnation time of mice. (e) Number of stagnation periods of mice. BA, biogenic amines; CAD, cadaverine; H, high; HIM, histamine; L, low; PEA, 2‐phenylethylamine; PUT, putrescine; TYM, tyramine. Values are expressed as mean ± SD (*n* = 10). *p < .05 and **p < .01 versus control group.

The concentration of BAs in Huangjiu was between 18.603 and 140.010 mg/L, and there were five types of BAs identified in Huangjiu, namely histamine, cadaverine, tyramine, putrescine, and phenethylamine (Wang et al., 2016). Moreover, the ethanol in the serum of mice peaked at 0.5 h and gradually metabolized within 2.5 h after gavage (data not shown). These results are consistent with previous studies (Guang, 2016). Subsequently, the effects of the five types of BAs on mice behavior (moving distance, average speed, moving time, stagnation time, and number of stagnation periods) were evaluated at 0.5 and 2.5 h postgavage. As shown in Figure 1a, after 0.5 h, the moving distance of the LHIM and HHIM groups was significantly reduced by 23.6% (*p* < .05) and 40.2% (*p* < .01), respectively, compared with the control group. In addition, the moving distance of mice in the HHIM group was significantly reduced by 36.3% (*p* < .01) 2.5 h after gavage, compared with the control group. In addition, the average speed of mice in the LHIM group and the HTYM group was significantly reduced by 21% (*p* < .05) and 22.5% (*p* < .05) 0.5 h after gavage, respectively. At 2.5 h postgavage, the average speed of mice in the HHIM group was reduced by 36.3% (*p* < .01). However, the average speed of the HTYM group did not show a significant reduction. Moreover, the total moving time is shown in Figure 1c. At 0.5 h postgavage, the moving time of mice in the LHIM and HHIM groups was reduced by 13.3% (*p* < .05) and 8.6%, respectively. However, after 2.5 h, the moving time of mice in the LHIM and HHIM groups did not change significantly. In addition to evaluating the behavior indicators above, the stagnation time and number of stagnation periods were also measured in this study. As shown in Figure 1d, the stagnation time of the LHIM (*p* < .01) and HHIM (*p* < .05) groups was longer than the control group at 0.5 and 2.5 h postgavage. In addition, the number of stagnation periods for mice in the LHIM and HHIM groups increased significantly (*p* < .05 and *p* < .01, respectively) compared with the control group at 0.5 and 2.5 h postgavage (Figure 1e). Interestingly, putrescine, 2‐phenylethylamine, cadaverine, and tyramine did not have significant effects on the stagnation time and the number of stagnation periods (*p* > .05) (Figure 1d,e). The results indicate that histamine, as the key component of BAs in Huangjiu, could significantly inhibit the behavior activity of mice by reducing the moving distance, average speed, and moving time, as well as increasing the stagnation time and the number of stagnation periods. These abnormal behaviors in mice are related to the phenomenon of hangover confusion and increased fatigue.

### 3.2 Influence of four brands of Huangjiu on mice behavior

**FIGURE 2:**
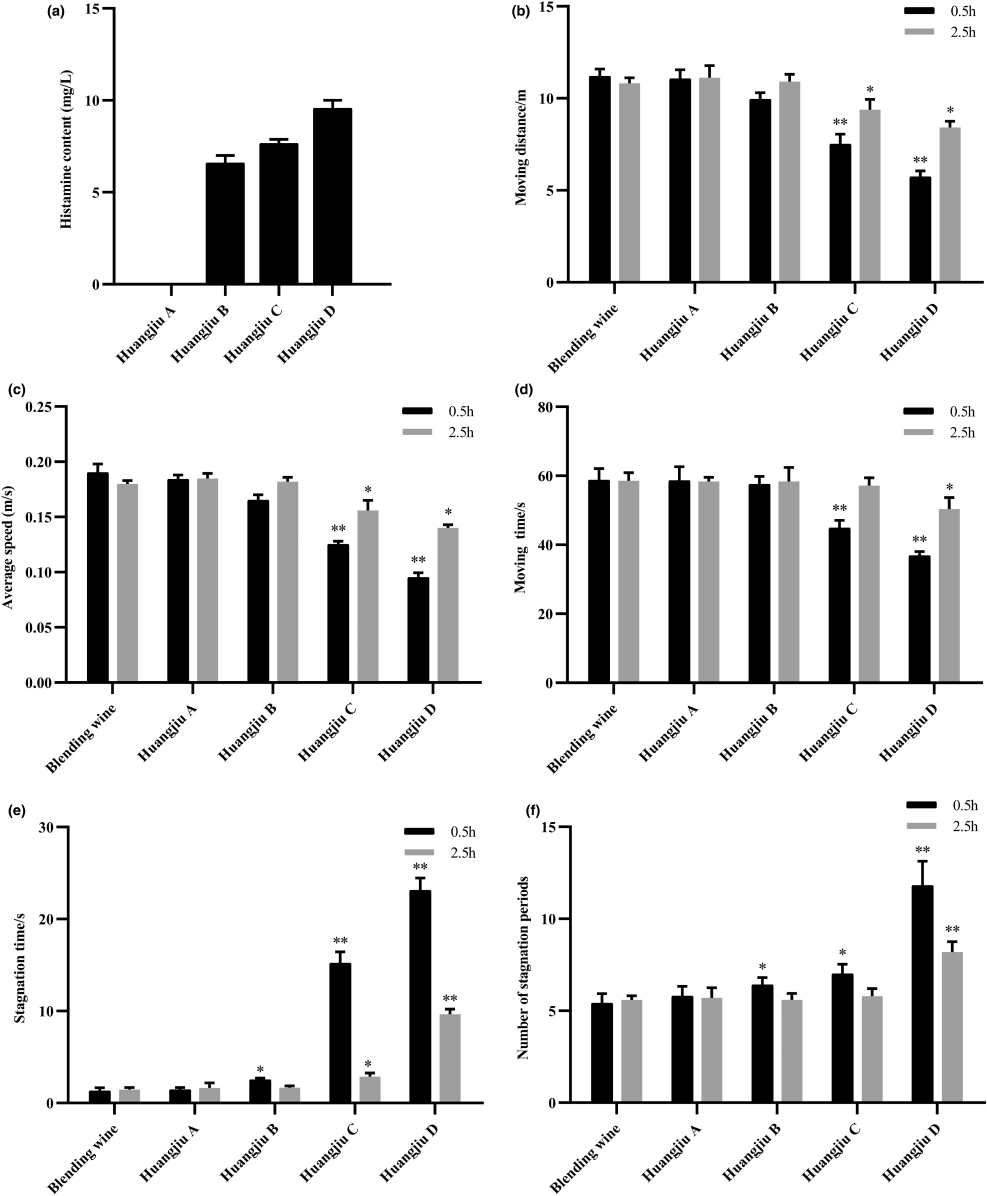
Concentration of histamine in four wine samples and the effects of different Huangjiu samples on mice behavior. (a) The concentration of histamine in four wine samples, (b) Moving distance, (c) average speed, (d) moving time, (e) stagnation time, and (f) number of stagnation periods. Values are expressed as mean ± SD (*n* = 10). *p < .05 and **p < .01 versus control wine group.

To understand the influence of histamine in Huangjiu on mice behavior, the effects of four of the most popular Huangjiu brands, designated as A, B, C, and D, on mice behavior were compared 0.5 and 2.5 h postgavage. First, the concentration of histamine in Huangjiu samples A, B, C, and D was measured by RP‐HPLC (Figure 2a). According to the Chinese National standard method, “Determination of Biological Amines in Food” (GB 5009.208–2016), the concentration of histamine in Huangjiu sample A was below the detection limit. Thus, it was used as a negative control in this study. Then, the hangover model was established to further explore the effect of histamine in Huangjiu on mice behavior.

As shown in Figure 2b, compared with the control wine group, the moving distance was significantly reduced in both Huangjiu C and D groups (*p* < .05 or *p* < .01). Moreover, the average speed and moving time of mice in the Huangjiu B, C, and D groups were significantly reduced (*p* < .05 or *p* < .01) (Figure 2c,d). Compared with the control wine group, the total number of stagnation periods and stagnation time of mice in Huangjiu B, C, and D groups dramatically increased (*p* < .05) (Figure 2e,f). These results reveal that Huangjiu A did not affect mice behavior significantly at 0.5 and 2.5 h postgavage. However, for Huangjiu samples B, C, and D, the behavior of mice at 0.5 or 2.5 h after gavage was significantly suppressed. Therefore, the following experiments were carried out with Huangjiu A as the negative control and base wine to determine histamine dosage.

### 3.3 Effect of histamine dosage on mice behavior

**FIGURE 3:**
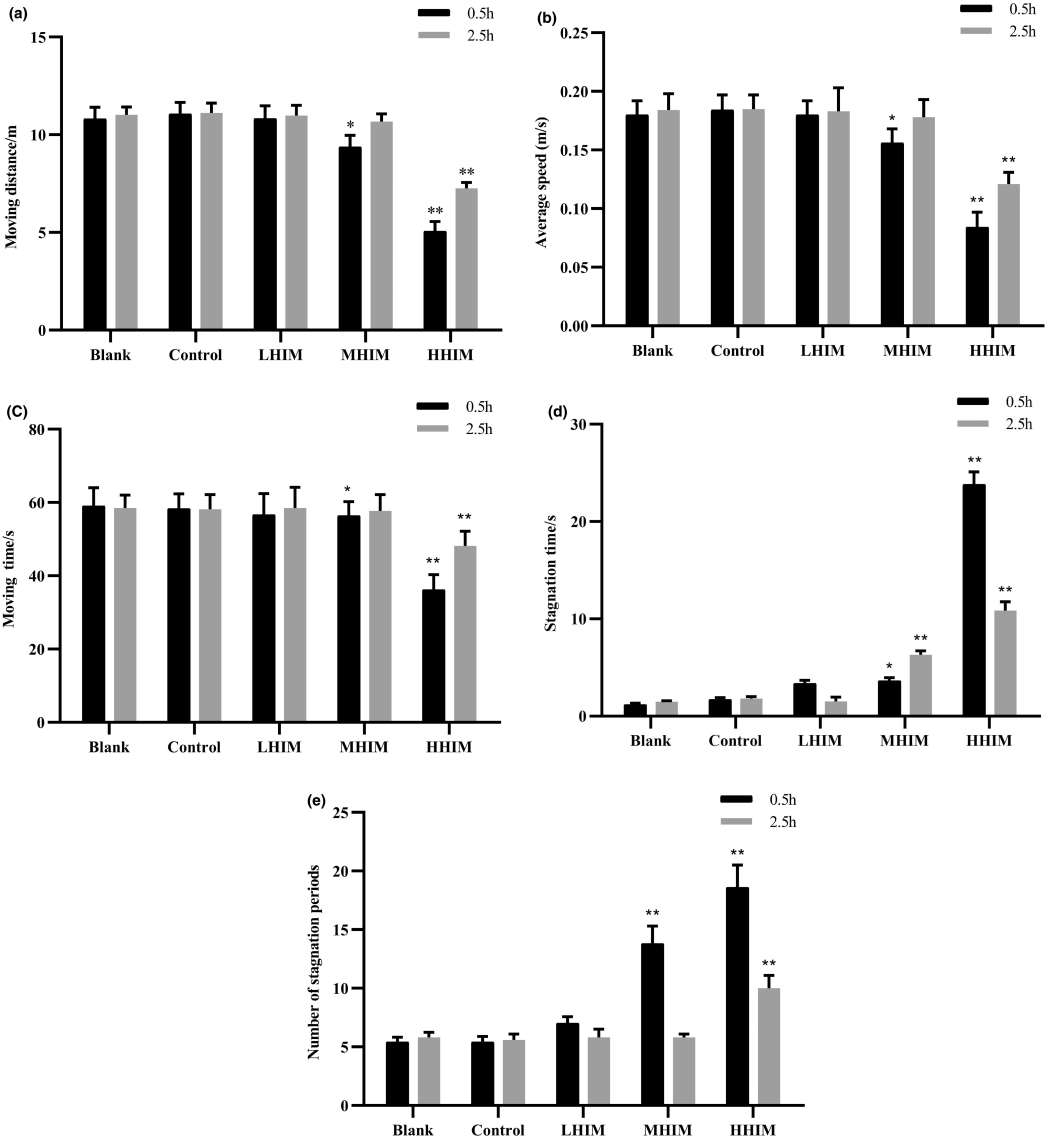
Effect of histamine concentration on mice behavior. (a) Moving distance, (b) average speed, (c) moving time, (d) stagnation time, and (e) number of stagnation periods. H, high; L, low; and M, middle. Values are expressed as mean ± SD (*n* = 10). *p < .05 and **p < .01 versus control group.

To reveal the effect of histamine on mice behavior, a certain amount of histamine (7, 35, and 69 mg/L) was added to Huangjiu A and administered to the mice. As shown in Figure 3, there was no significant difference between the blank group and the control group (*p* > .05). This result is consistent with Figure 2. At 0.5 h, compared with the control group, the moving distance of mice in the MHIM group decreased significantly by 8.8% (*p* < .05), but it was not significantly different (*p* > .05) at 2.5 h. However, the moving distance of the HHIM group decreased remarkably by 50.7% and 32.9% (*p* < .01) at 0.5 and 2.5 h postgavage, respectively (Figure 3a). At 0.5 h, compared with the control group, the average speed and moving time of mice in the MHIM group significantly decreased by 8.2% and 5.1% (*p* < .05), respectively, and the stagnation time and number of stagnation periods significantly increased by 47.7% (*p* < .05) and 86.5% (*p* < .01), respectively. Moreover, at 2.5 h, compared with the control group, the average speed and moving time significantly decreased (*p* < .01), and the stagnation time and number of stagnation periods significantly increased (*p* < .01) in mice of the HHIM group (Figure 3b–e). Therefore, these behavior changes indirectly suggest that histamine causes dizziness, a decrease in motivation, or fatigue in mice when its content reaches 35 mg/L.

### 3.4 Effect of histamine on the content of 5‐HT, 5‐HIAA, DA, and NO in brain tissue of mice

**FIGURE 4:**
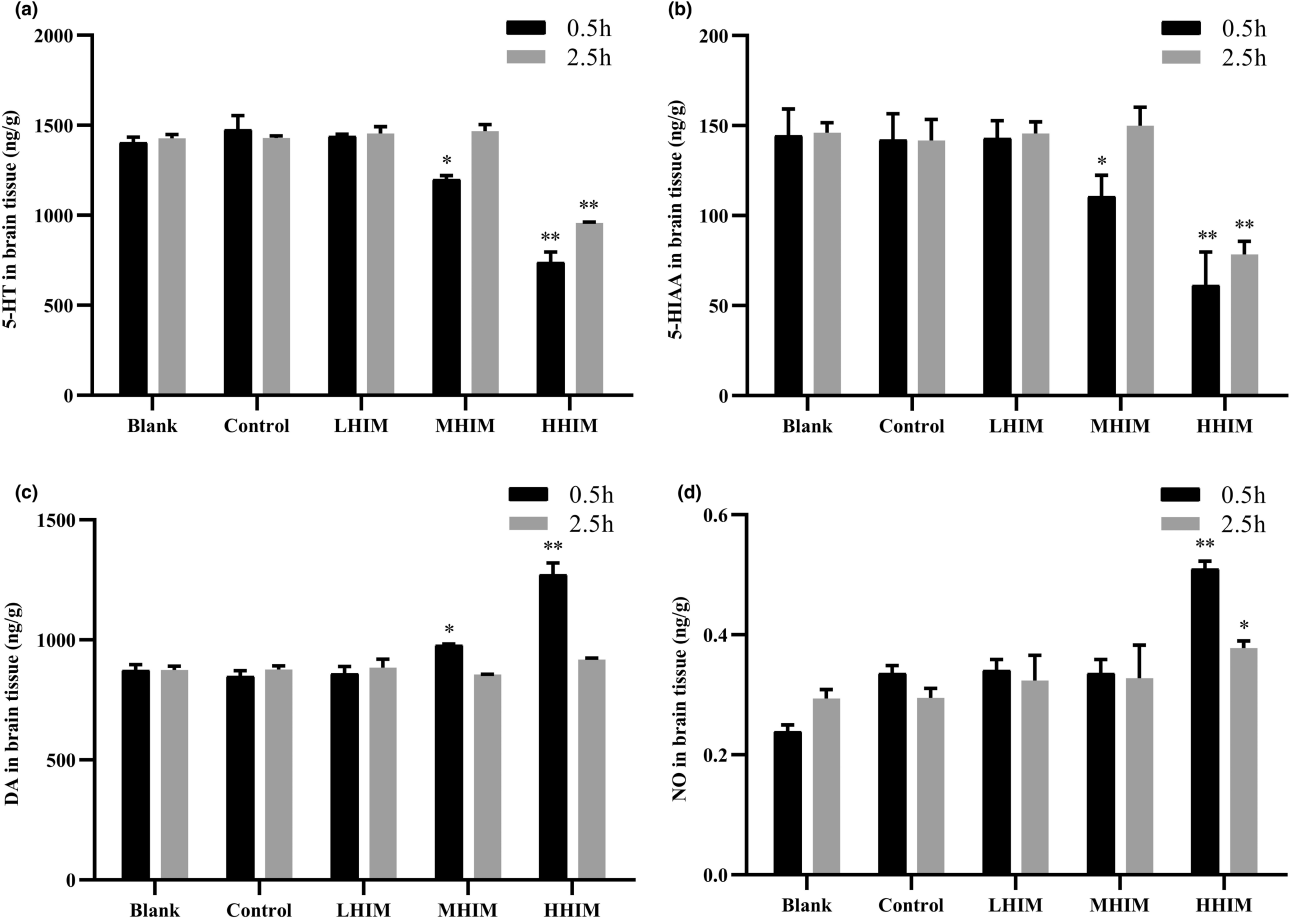
Content of 5‐HT, 5‐HIAA, DA, and NO in brain tissue. (a) The concentration of 5‐HT in mouse brain tissue (*n* = 10). (b) The concentration of 5‐HIAA in mouse brain tissue. (c) The concentration of DA in mouse brain tissue. (d) The concentration of NO in mouse brain tissue. Values are expressed as mean ± SD (*n* = 10). *p < .05 versus, **p < .01 versus control group.

5‐HT is a mediator of target cells and a receptor in the process of vasoconstriction and relaxation, thus, a disorder in 5‐HT metabolism is the basis for the onset of migraine (Wang et al., 2018). In this study, the effect of histamine on the content of neurotransmitters related to headaches, including 5‐HT, 5‐HIAA, and DA, was evaluated in the brain tissue of mice. In addition, NO, which could prolong the state of a migraine, was also measured. As shown in Figure 4a, compared with the control group, there was no significant difference in the content of 5‐HT in the brain tissue of the mice in the LHIM group at 0.5 and 2.5 h. However, the MHIM group was decreased by 18.0% (*p* < .05) at 0.5 h. At 0.5 and 2.5 h, the content of 5‐HT in brain tissue of the HHIM group decreased remarkably by 49.4% and 32.8% (*p* < .01), respectively.

The content of 5‐HIAA in the brain tissue showed a similar trend as 5‐HT. The MHIM and HHIM groups exhibited decreases of 22.3% (*p* < .05) and 57.1% (*p* < .01) at 0.5 h, respectively, compared with the control group. The HHIM group exhibited a significant decrease of 45.4% at 2.5 h, as shown in Figure 4b.

The contents of DA in the brain tissues of mice from each group are shown in Figure 4c. Compared with the control group, there was no significant difference in the LHIM, MHIM, and HHIM groups (*p* > .05) at 2.5 h. However, the content of DA in the MHIM and HHIM groups increased by 6.6% (*p* < .05) and 46.2% (*p* < .01) at 0.5 h, respectively. These changes indicate that the content of DA increased as a result of the high amount of histamine over a short period of time.

The contents of NO in the brain tissues of mice from each group are shown in Figure 4d. Compared with the control group, the content of NO in the brain tissue of mice in the LHIM and MHIM groups had little change at 0.5 h. However, the content of NO significantly increased by 36.7% (*p* < .05) and 8.6% (*p* < .05) in the HHIM group at 0.5 and 2.5 h, respectively. The increase in NO after gavage suggests that inflammation reactions occurred in mice.

### 3.5 Effect of histamine on the mRNA expression level of *Htr1a*, *Htr1f,* and *Htr2c* genes in brain tissue

**FIGURE 5:**
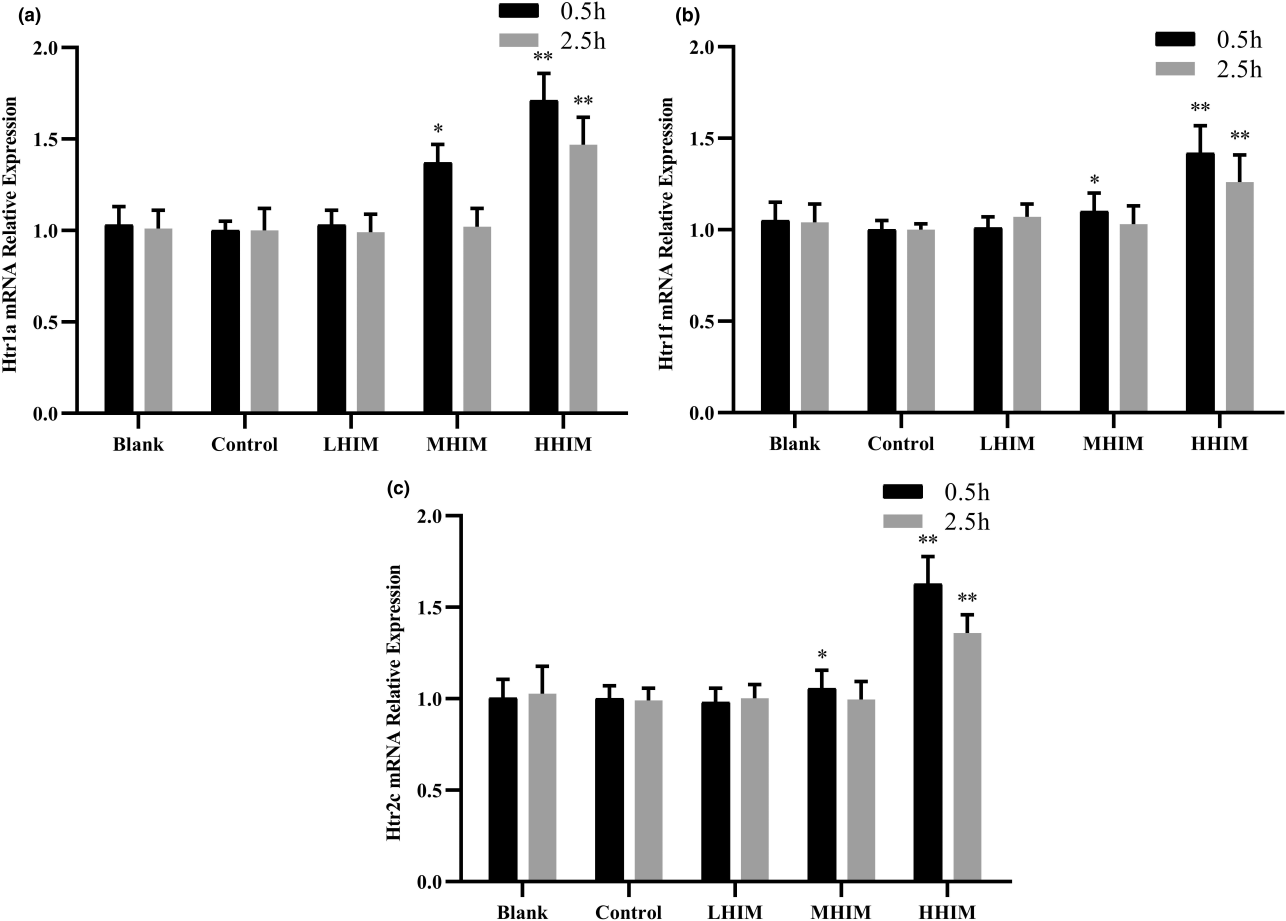
Effects of histamine on the relative mRNA expression. (a) Expression of *Htr1a*. (b) Expression of *Htr1f*. (c) Expression of *Htr2c*. Values are expressed as mean ± SD (*n* = 10). *p < .05 versus control group, **p < .01 versus control group.

5‐HT plays an important role in regulating pain by binding to various receptors. Therefore, the key genes related to 5‐HT receptors, *Htr1a*, *Htr1f*, and *Htr2c*, were detected in this study. These genes play essential roles in regulating various behaviors and mental activities (Gruber et al., 2010). As shown in Figure 5, compared with the control group, the expression levels of *Htr1a*, *Htr1f*, and *Htr2c* genes in the brain tissues of mice did not change in the LHIM group (*p* > .05), indicating that this dosage of histamine could not activate or inhibit the expression levels of these three genes. Furthermore, the expression levels of *Htr1a*, *Htr1f*, and *Htr2c* were increased by 27.0%, 10.1%, and 8.5% (*p* < .05) at 0.5 h in HHIM group, respectively. However, the expression level was not significantly different between the three histamine groups and the control at 2.5 h. Thus, when the histamine content reached 35 mg/L, the expression of related genes was activated; however, after 2 h, the expression of these genes returned to a normal level. The results indicate that when the histamine content reached 69 mg/L, the expression of related genes did not return to its original level in a short time period.

## 4 DISCUSSION

As a traditional Chinese wine, the undistilled product, Huangjiu, contains more bioactive substances than other distilled wines, such as Chinese Baijiu (Jin et al., 2021). Some substances found in Huangjiu, such as nutrients and functional ingredients, have health benefits, while others are associated with hangover symptoms, such as head neuralgia, rapid heartbeat, and behavioral disorders (Bach et al., 2012; Broadley, 2009). Zhong et al. (2012) determined BAs in semisweet Chinese Huangjiu from the Shaoxing region and found that putrescine, tyramine, cadaverine, and histamine were the prominent BAs. Using the pharmacokinetics parameters, Peng et al. (2019) discovered that tyramine, cadaverine, and histamine have slower elimination rates in the human body and slow down the metabolism of ethanol for a long period of time. These BAs had side effects on human health, which were directly related to headache, vomiting, dizziness, thirst, and other symptoms. However, there are no studies on the specific substances in Huangjiu that cause discomfort and their related mechanisms.

In this study, behavioral parameters, such as movement and stagnation in mice, were used to screen for specific BAs in blended wine that may affect the behavior of mice and hangover headache‐related indices. The results of mice behavior show that among the five BAs, histamine in blended wine had the most obvious inhibitory effect on the behavior of mice (Figure 1). Previous studies also showed the metabolic rate of histamine in the human body was lower than that of other substances in Huangjiu, and it could slow down the metabolism of ethanol. Thus, histamine is more likely to cause headaches (Zhang et al., 2015). Therefore, in this study, histamine was selected as the key BA, and we further explored its effects on the behavior of mice with acute alcoholism and hangover headache‐related indices.

Except for the blended wine samples, four brands of commercial Huangjiu were used in this study so the results could be used for reference to evaluate the actual samples and rule out the influence of histamine. Therefore, four brands of Huangjiu were selected from the Shaoxing region because this region is well known for Huangjiu brewing in China. The histamine content in Huangjiu A was below the detection limit, according to the Chinese National Standard. Moreover, upon ingestion, it did not inhibit the behaviors of mice (Figure 2). Therefore, Huangjiu A was used as the negative control group in the following studies to establish the hangover mice model. The results also confirmed that histamine in Huangjiu had a significant effect on the behavior of mice in the subsequent experiments (Figure 3). Histamine is involved in local immune and inflammatory responses, and it performs various functions as a neurotransmitter (Benetti et al., 2015; Cataldi et al., 2014). A previous study demonstrated that human intake of 8–40 mg of histamine caused dizziness, headache, and diarrhea (Doeun et al., 2017). In this research, when the content of histamine reached 35 mg/L, the content of 5‐HT and 5‐HIAA decreased, and the content of DA increased significantly at 0.5 h postgavage (Figure 4a–c). 5‐HT is a monoamine neurotransmitter in the human central nervous system and has a wide range of effects on organisms (Kilinc et al., 2016). At present, there are numerous reports on the therapeutic effects of 5‐HT receptor agonists and antagonists on neuropathic pain. The lower the content of 5‐HT, the more pronounced the migraine will be (Wallinga et al., 2011). 5‐HIAA is formed by 5‐HT under the catalysis of monoamine oxidase (Barnes & Sharp, 1999). As the metabolic end‐product of 5‐HT released from the central nervous system, 5‐HIAA reflects the level of 5‐HT metabolism. Studies have shown that the content of 5‐HIAA in a migraine mice model was lower than that in normal mice (Ma et al., 2010). In this research, the content of 5‐HIAA in the brain tissue of the mice in the MHIM and HHIM groups was significantly lower than that in the blank and control groups, which is similar to previous research. DA is a catecholamine neurotransmitter, and it may be involved in the occurrence of migraine through the trigeminal pathway (Wang & Shen, 2017). In this study, the content of DA showed an upward trend with the increase in histamine content (Figure 4c), which indicates that high content of histamine may cause migraines. In addition, as a relaxing factor of vascular endothelium, NO not only stimulated migraine headaches but also prolonged the effects (Gruber et al., 2010). Compared with other groups, the content of NO in brain tissue significantly increased in the HHIM group (Figure 4d). These results indicate that histamine in Huangjiu caused headaches and behavioral changes in mice when the content of histamine in Huangjiu reached 35 mg/L. Moreover, when the histamine content exceeded 35 mg/L, the symptoms, such as headache, dizziness, headache, and behavior disorders, were aggravated.

The mechanism of 5‐HT played a role in pain modulation by binding to multiple receptors (Wang et al., 2018). The expression of 5‐HT receptor genes, *Htr1a*, *Htr1f*, and *Htr2c*, was found to be activated in this study and contributed to headaches and behavioral inhibition in mice (Figure 5).

Australia and Switzerland have stipulated that the histamine content in grape wine should not exceed 10 mg/L. In France, the content must not exceed 8 mg/L, and in the Netherlands, the content must not exceed 3.5 mg/L (Lehtonen, 1996). At present, China does not stipulate the limit of histamine content in grape wine or Huangjiu. The content of histamine in Chinese Huangjiu is between 5.02 and 78.5 mg/L (Lu et al., 2007), which is much higher than grape wine. Therefore, exploring the source of histamine and formulating strategies to control its content could improve the safety of Huangjiu.

In summary, high content of histamine had stronger and longer inhibitory effects on mice. However, the specific mechanism of inhibition on mice behavior requires further investigation. When the mice dosage was converted to the human dosage, histamine content of 35–69 mg/L in Huangjiu may lead to adverse symptoms and behavior in humans. This may provide relevant reference data for the Huangjiu production industry to improve the safety of consumption. Therefore, based on our results, the histamine content in Huangjiu should be strictly controlled in the process of production.

## FUNDING INFORMATION

This work was supported by the National Key Research and Development Program of China (grant number 2021YFE0192000).

## CONFLICT OF INTEREST

The authors have declared that there is no conflict of interest.
